# Notes

**Published:** 1902-12

**Authors:** 


### NOTES.

Dr. E. M. Ranck, of the H. K. Mulford Co., Glenolden, Pa., has inaugurated a series of experiments and investigations relative to the organisms giving rise to distemper or rhinoadenitis, ship-fever, and influenzoid conditions among horses shipped from the Western sales centres.

"Some of the Difficulties in Introducing Improved Live-stock Into the State" was the subject of a paper before the Louisiana State Breeders' Association by Dr. W. H. Dalrymple in April, 1902.

For more than fifty years the *Physicians' Visiting List* has been published by the well-known medical book publishers, Messrs. P. Blakiston's Son & Co., of Philadelphia, Pa. The valuable tables it contains for ready reference, and the fact that it is a complete record book for daily practice, and so compact in form, have added each year to its popularity and testimonials as to its worth.

